# Clarification of ethical principle of the beneficence in nursing care: an integrative review

**DOI:** 10.1186/s12912-023-01246-4

**Published:** 2023-03-30

**Authors:** Rozita Cheraghi, Leila Valizadeh, Vahid Zamanzadeh, Hadi Hassankhani, Anahita Jafarzadeh

**Affiliations:** 1 Medical Surgical Nursing Department, School of Nursing and Midwifery, Tabriz University of Medical Sciences, Tabriz, Iran; 2 Road Traffic Injury Research Center, Faculty of Nursing and Midwifery, Tabriz University of Medical Sciences, Tabriz, Iran; 3 Student Research Committee, School of Nursing and Midwifery, Tabriz University of Medical Sciences, Tabriz, Iran; 4 Department of Pediatric Nursing, School of Nursing and Midwifery, Shahid Beheshti University of Medical Sciences, Tehran, Iran; 5 Department of Medical Surgical Nursing, School of Nursing and Midwifery, Shahid Beheshti University of Medical Sciences, Tehran, Iran

**Keywords:** Beneficence, Ethic, Nursing, Care, Integrative review

## Abstract

**Background:**

Ethics-based nursing practice can transform health care practices. As the biggest human capital in the health care system, nurses are obliged to follow ethical principles in this field. One of these ethical principles; is beneficence, which is considered the core of nursing care. This study aimed to investigate clarification of the principle of beneficence in nursing care and its related challenges.

**Methods:**

This integrative review was conducted using the Whittemore & Knafl method in 5 stages, including problem identification, searching the literature, evaluating primary sources, analyzing data, and presenting the results. Databases like SID, Irandoc, Magiran, Google Scholar, Web of Science, PubMed, and Scopus were searched using the keywords; “beneficence”, “ethic”, “nursing” and “care” in English and Persian in the time range of 2010 to 10 February, 2023. After applying inclusion criteria and assessing the articles using Bowling’s Quality Assessment Tool, finally, 16 papers were included from 984.

**Results:**

After reviewing and evaluating the qualified articles, the findings were classified into four main categories: (1) nature, (2) applicability, (3) Relevant and influential factors, and (4) challenges related to the ethical principle of beneficence in nursing care.

**Conclusion:**

Based on the results of this review it seems that paying attention to clarification the principle of beneficence in nursing care can provide positive outcomes for patients to benefit from this principle and finally, it leads to increasing the well-being and health of patients, reducing their mortality rate, increasing satisfaction and maintaining the respect and human dignity of patients.

## Background

Today, nurses are considered the biggest human capital in the health care system [1]; so the care provided by them is one of the main components of services in this system [2]. Providing care based on ethics is one of the approaches in the nursing discipline that nursing ethics theorists have discussed [3]. An ethical nursing practice can transform healthcare practices [4]. Nursing ethics is considered an issue of bioethics, and a short time has passed since its formation [5]. Bioethics is the application of ethical theories and principles in ethical issues or healthcare dilemmas [6]. Therefore, understanding these principles and values is considered the first step in understanding ethics and its relationship with health care [7]. Good nursing care aims at the enhancement of the dignity of the human being in all dimensions and also succeeds to realize this intention in practice and it is considered dignity-enhancing care [8]. Hence, The nursing profession is considered a discipline with a health-oriented approach, which, focuses on the caring situations and helps people to adapt to their disease and improve their current performance capacity despite the disease [4].

The history of nursing shows that professional ethics, as a familiar concept in nursing, has been an inherent part of this profession since the early days, and it is internationally recognized as an essential part of nurses’ practice [9]. Four basic principles for bioethics were stated by Beauchamp and Childress, which are autonomy, justice, beneficence, and non-maleficence [10]. Ideally, these principles are the same ones that all nurses should be aware of in their practice and follow in caring for clients in order to provide the best, safest, and most humanistic care for all of them [11]. One of these basic principles is “beneficence” which is considered the basis of nursing and medical activities that performing such beneficial actions can encourage the motivation of good works [7]. It dares to say that the ethical principle of beneficence is an integral part of the mission of the nursing profession [12], which, according to a definition, implies doing positive things to help patients [7]. “Beneficence” is mentioned as a key component of bioethics and its outcome can help to receive the important and legitimate interests of the patient [13]. Although the concept of beneficence is widely used in medical sciences, it is not easy to define it precisely [14], and even few studies in health sciences have addressed the concept of beneficence [15–17]. In the nursing discipline, although professional ethics are well established, there is no real understanding of ethical principles [9], for this reason, several interpretations and definitions have been proposed [14]. Various definitions of beneficence have been presented in different kinds of literature, which consider this principle as the essence of ethics and doing “good” with mercy, kindness, generosity, and charity for patients [10]. Also, sometimes this principle is also mentioned in the meaning of doing good, kindness, and charity or any action that benefits others [14]. Singh & Ivory (2014) consider beneficence as the responsibility of healthcare experts to promote the well-being of patients through research and implementation of therapeutic interventions with the highest probability of positive patient outcomes [15]. In addition to these definitions, the principle of beneficence emphasizes the ethical commitment to the benefit, including protecting patients’ rights, preventing harm to them, and helping those at risk [18]. In another view, beneficence is interpreted as ensuring the provision of care with positive benefits and protecting patients, which plays a major role in all health care [19]. These views confirm the difficulty of implementing the principle of beneficence, which requires the definition of “what is good for the patient“ [20].

On the other hand; studies show that beneficence as an integral part of bioethics has always been beside other ethical principles, including autonomy, justice, and confidentiality, and therefore, there is a major challenge to balance the patient’s right to choose and the benevolent intention of the caregiver [21], and as mentioned, sometimes the care measures that guarantee beneficence may violate other ethical principles such as patient autonomy [22]. Of course, ethical conflicts are an integral part of nursing, because they try to fulfill their needs, goals, and efforts in the overall provision of nursing care [23], but despite this point of view, it should be said that nurses’ ethical commitment requires benefiting the patient by ignoring personal interests for the needs of the patients, their well-being and preferences [24, 25]. It has been found that ethical dilemmas are sensitive to culture and context and reflects differences in culture, social attitudes, and legal principles [23], studies also emphasize that in order to beneficence in nursing care, they should pay attention to the cultural, social and ethical factors of patients [26].

What is obvious is that the basic issue in the principle of beneficence is the commitment to the benefit of patients in nursing care [27]. Although various studies have addressed this concept in a limited way, very few and scattered studies have focused on bioethics in nursing sciences and the principle of beneficence, and there is still no comprehensive and clear understanding of this concept as an ethical principle in providing nursing care. It should be noted that an integrative review study is a specific review method that summarizes empirical or theoretical studies that have already been conducted to provide a more comprehensive understanding of a specific phenomenon or healthcare problem [28]. Based on this, integrated reviews have the potential to expand the body of knowledge and create nursing science, knowledge, research, practice, and policies. In addition, these kinds of studies show the current state of knowledge in each discipline, help to develop theory, and have a direct application in the clinical fields and health policies [29]. Therefore, it seems that the results of this study can help clarify the concept of beneficence in nursing. Considering the importance of the principle of beneficence in nursing care and increasing patient satisfaction, we decided to review the studies conducted on this issue.

## Methods

### Study design

This study is an integrated review based on articles related to the ethical principle of beneficence, which was conducted to collect data from various studies on this concept. This integrative review was conducted using the Whittemore & Knafl method in 5 stages of review, including (a) problem identification, (b) searching the literature, (c) evaluating data from primary sources, (d) analyzing data, and (e) presenting the results, using of this method also increases the rigor of this study [29–31].

### Search strategy

According to the Whittemore & Knafl method, a) in the first stage, the following question was set to answer the study’s aim: What is the ethical principle of beneficence in nursing care and its related challenges?

b) In the second stage, searching for articles was done by two researchers in the time range of 2010 to February 10, 2023. Databases like SID, Irandoc, Magiran, Google Scholar, Web of Science, PubMed, and Scopus were searched using the keywords; “beneficence,“ “ethic,“ “nursing,“ and “care” in English and Persian separately or combined using Boolean operators: AND and OR for papers that were published. The result of the initial and comprehensive search included 984 articles. After applying the inclusion criteria, including access to the full text of the article, writing in Persian and English, and the presence of keywords in the title and abstract of the article, finally, 968 were removed.

### Eligibility criteria

**Fig. 1:**
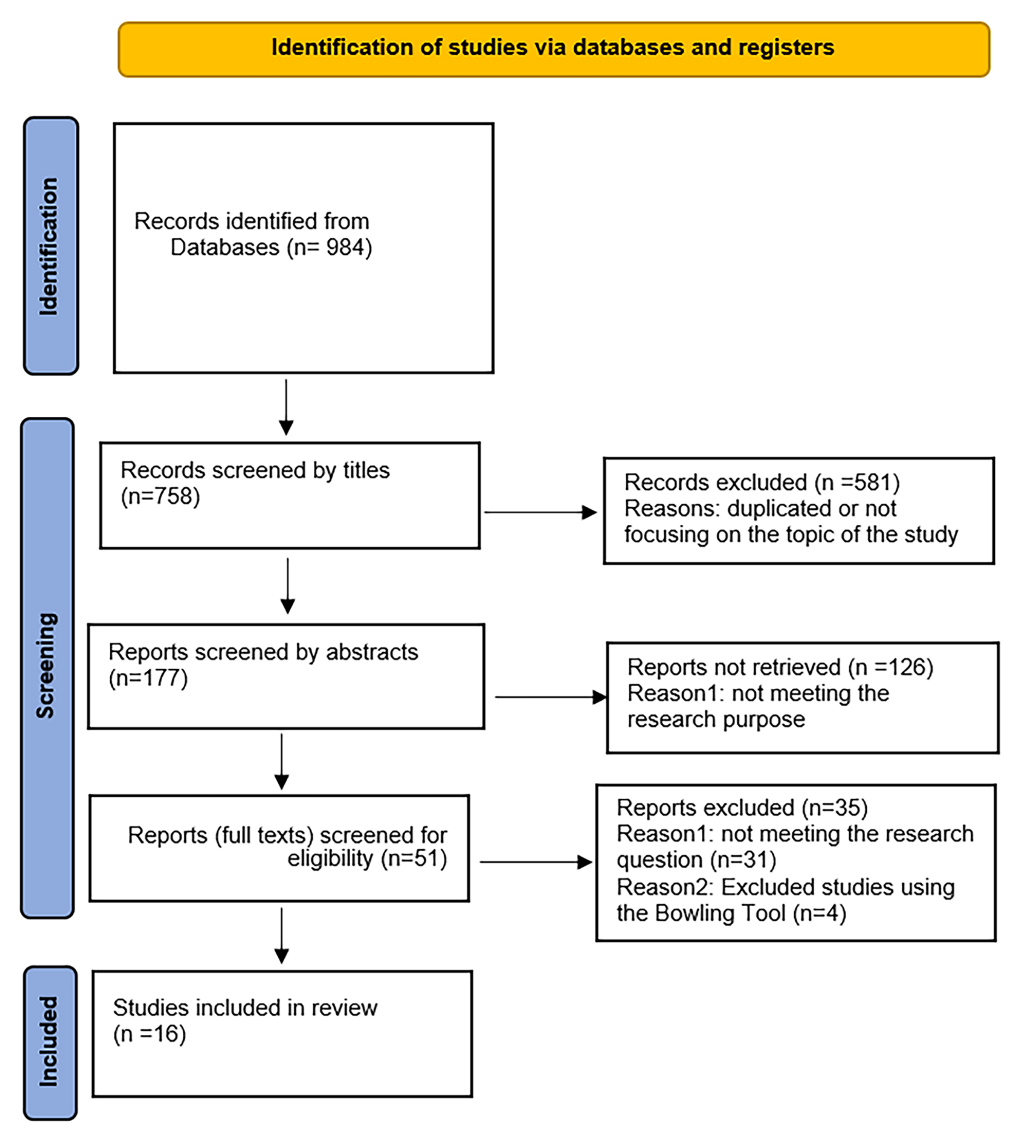
PRISMA flow diagram

c) In the third stage, to evaluate the data, two researchers reviewed the content of the studies to evaluate their quality using Bowling’s Quality Assessment Tool [32], so 4 articles were excluded by this tool, and then the results were compared. Finally, 16 articles were included in this study (Fig. 1 following the renewed PRISMA guideline) [33]. The Bowling tool consists of items that were used to check the structure of the methodology and present the results of the studies with a triple scale: yes, weak and unreported, which led to the elimination of weak and unreported articles.

### Data extraction

The key data extracted from the articles included: the authors’ profile, the publication’s year, the country of the study, the title and the methodology of the study, and also the main results that were extracted by the two researchers.

### Data synthesis

Data charts were designed independently by two researchers and they were evaluated. The data chart was updated in a continuous process. Then, the extracted data were analyzed and interpreted. The data analysis process was initiated when the final 16 papers for inclusion were determined and had been verified by all three authors. First, a data extraction sheet were created to document the required information for each review, including the five methodological stages. So, each review was analyzed individually by two researchers separately. All of the data gathered in the data extraction sheets was read by the same two researchers, who reached a consensus on selecting pertinent and significant items for each stage. Then, the similarities and differences among the reviews with regard to each methodological stage were examined throughout the data analysis process. Finally, conclusion drawing and verification were performed by all three authors to ensure that all 16 reviews were thoroughly assessed in terms of methodological stages and that the results described matched the research questions of this present study.

### Quality appraisal

Whittemore and Knafl (2005) state that assessing the quality of the included evidence is not essential in a supplementary review [30]. All studies meeting the inclusion criteria, regardless of their methodological quality, were retained in the review to examine all evidence of the factors that influenced the nursing role implementation in practice settings.

## Results

According to the framework of review and selection of articles, 16 articles were selected from 984 articles. All articles included in this study were in English, including quantitative (n = 2) and qualitative (n = 14) studies.

After reviewing and evaluating the qualified articles, the findings were classified into four main categories as follows: (1) nature, (2) applicability, (3) Relevant and influential factors, and (4) challenges related to the ethical principle of beneficence in nursing care (Table 1).

**Table 1:** Main categories and sub-categories extract from the review of selected articles

Main categories	sub categories
**Nature**	- doing “good” [12, 20, 22, 26, 34–37] - Maximum positive benefits for patients[16, 20, 22, 24, 34, 36–38] - Best care[16, 23, 34, 35, 37] - The core of care[20] - Ethical commitment to the benefit of patients[39]
**Applicability**	- No harm to the patient[non-maleficence][12, 20, 22, 26, 34–38, 40] - Improving the well-being of patients[20, 22, 24, 25, 37, 38] - Attention to patient’s preferences and needs[16, 25, 34, 41] - justice-fair distribution of resources[20, 24, 35] - Predicting and reducing pain[12, 41] - Palliative and end-of-life care[38, 41] - Patient Safety[24, 37] - Health Promotion[22, 34] - Patient support[16, 34] - Respect for human dignity[38] - Truth-telling[26] - Informing the patients[16] - Applying knowledge for the benefit of patients[24] - Decision-making in controversial situations[36]
**Relevant and influential factors**	- Cultural, social, and ethical differences of patients[16, 26] - Knowledge, ethical insight, and commitment in nursing care[16, 22, 42] - Organizational ethical climate in the implementation of beneficence [37]
**Challenges**	- Lack of acquainted with the definition of beneficence concept[42] - Applicability of beneficence[24, 25, 34] - Conflict between beneficence and preservation of patient autonomy[16, 22, 25, 26, 34, 37–39] - The conflict between beneficence and justice in care[24, 34, 38]

## Discussion

The present study investigated the ethical principle of beneficence in nursing care and its related challenges in different studies. This integrated review identified four main categories (nature, applicability, relevant and influential factors, and challenges) and twenty-six sub-categories. It is obvious that the ethical principle of beneficence is an integral part of the nursing code, also called the “moral heart of the nursing profession,“ so a nurse should provide nursing care only with the goal to provide benefit to the client [16]. It is on this basis in nursing ethics, beneficence is considered a moral commitment that is worthy and generous [43]. Despite the role of this ethical principle in nursing care, the Lack of acquaintance with the definition of the beneficence concept is considered an important challenge [36]. What is observed in the reviewed studies; “doing good” is considered beneficial [12, 20, 22, 26, 34, 35, 36, 37]. The difficulty of implementing this ethical principle; requires a clear definition of “what is good for a patient“ [20]. This definition has been expressed in different forms in different studies, including doing good [12, 20, 22, 26, 34, 35, 36, 37], maximum positive benefits for patients [16, 20, 22, 24, 34, 36, 37, 38], best care [16, 23, 34, 35, 37], the core of care [20], and ethical commitment to the benefit for patients [39].

The patient’s best interest must be the center of ethical decision-making at all times [44], so healthcare workers have to provide more patient-centered care to include attention to patient priorities and preferences [12]. It is on this basis that in every context or every part of society, people should be encouraged to do good acts and benevolence, and their work and actions, both professional and personal, should benefit the people of the whole society [45]. The outcome of this good action will be providing maximum positive benefits for the patients and achieving the best care for them [34, 35, 46], which will ultimately improve their health and general well-being [45, 46] because the core of ethical care is the pursuit of moral attitude and goodness [47].

A beneficent act promotes the betterment and welfare of patients, and nurses should deliver professional care to enhance the recovery of patients. As Beauchamp and Childress (2013) mentioned this principle is positive beneficence and break it down into three components: to prevent evil or harm; to remove evil or harm; and to do or promote good [48, 49, 50]. Also, beneficence considers the balancing of the benefits of treatment against the risks and costs; so, the healthcare professional should act in a way that benefits the patient [51], and Edwards (2009) states that beneficence is about promoting the well-being of those with whom we interact [52]. In most cases, the principle of beneficence and non-maleficence mean together [12, 16, 22, 26, 34, 35, 36, 37, 38, 40, 44], because non-maleficence prevents harming the patient but rather seeks to improve people’s well-being and health [20]. In fact, avoiding any useless intervention and any action that may lead to irreparable harm (such as death) to the patient; is interpreted as non-maleficence. As mentioned, beneficence and non-maleficence are often two sides of the same coin and are discussed together, as the former involves doing good acts that benefit others, and the latter involves avoiding actions that harm oneself or others. Having these two principles together, conflicts between the care team and the patient’s families are inevitable [53]. Although both of these principles are important, the duty of non-maleficence is considered a stronger commitment in health care [54], and the principle of beneficence requires that “good” be done, and “harmful” actions for patients be avoided [10]. It may be said that the distinction between these two important ethical principles lies in the fact that beneficence is an ethical commitment to take positive steps to help patients and not merely to prevent harm [55].

Recent research shows that healthcare institutions and organizations that have a benevolent ethical atmosphere may achieve better clinical results, improve patient and family satisfaction, and reduce patient mortality [56]. The importance of these two principles, as the main values of nursing, always influences their behavior to direct their actions for the benefit of patients [36]. Nevertheless, nurses should understand that benevolent actions may not always be a benefit to patients [57] and sometimes conflict with preserving the ethical principle of autonomy [16, 22, 25, 26, 34, 37, 38, 39]. Per Ross’s ethical principles, all human beings should follow before the factors such as the benefit or utility of outcomes and results, like justice-fair distribution of benefits, risks, and costs [58].

Since the professional duty to respect the rights of patients and their autonomy in decision-making is considered a universally accepted norm [57], it may be said that nowhere in ethical decision-making situations; the contradiction is not as obvious as when the principles of beneficence and autonomy collide [46]. However, in all clinical decision-making situations, novice and experienced nurses must consider the patient’s right to autonomy and consider their nursing care beneficial for the patient’s health outcomes [59]. To achieve this level of care, nurses should expand the scope of ethical principles of beneficence, autonomy and, patient Advocacy in patient care [22].

The principle of beneficence makes it necessary to provide equitable care based on need and equally without discrimination for all patients [53], so when nurses are required to make decisions that negate the patient’s autonomy, these decisions should be considered as complying with the principle of benefit for them [59]. When beneficence and autonomy are in conflict and coercion is necessary for beneficence, the healthcare team needs a way to resolve this dichotomy and retain their self-esteem, in these cases, they may experience cognitive dissonance, a phenomenon that defines the mental tension arising when conflicting attitudes are held or when behaviors are incompatible with certain attitudes [60]. So, ignoring the principle of patient autonomy is considered a very serious issue, but it can be considered when there are real reasons to maximize beneficence and as long as it is consistent with the patient’s values [46, 61]. Therefore, a nurse can only prioritize beneficence over the principle of client autonomy when there is a good reason that the client’s respect will be preserved and she/he will really benefit from this approach [16, 39]. Nevertheless, sometimes nurses report that they experience ethical dilemmas in ethical principles; in these cases, it is recommended that nurses decide on the best care option for patients; consider their needs and preferences [25]. The beneficence can include patient autonomy because “the best interests of patients are closely related to their preferences,“ which is one of the main duties of the health team towards all patients [62]. However, beneficence is defined as ‘the principle of doing good and providing care to others’ by Berglund(2007) [63].On the other hand; respecting the needs, values, and preferences of the patient and their family are at the core of nursing care, and nurses should be aware of these needs by documenting the life history of the patients because the needs and interests of patients are formed based on religious, cultural and social issues [16, 26, 41, 64]. This attention to values in care protects and defends the client and ensures that the best decisions are made in the best way [64].

A concern to promote beneficence may be expressed in traditional medical ethics by paternalism, where the health professional makes a decision based upon a perspective of acting in the patient’s best interests. However, some believed that this approach acts against person-centered values found in nursing ethics [65]. By the way, health care balances the ethical principles of beneficence and justice, and the best possible care for patients with equitable care is presented for the entire population using limited resources in the most efficient way [35]. It should also be considered that the principle of justice provides a key link between the importance of health for individuals and the responsibilities of promoting the health of society [66]. Indeed, it is optimal to both respect choice and protects from harmful choice, but sometimes this is not possible [49]. However, any choices for the patient may sometimes create a conflict between justice and beneficence [34]. Some researchers consider the attention to beneficence before justice, although the principle based on justice is based on profit, which is reflected in the health system [66]. However, the priorities of health care should be focused on distributive justice, which means that decisions should be made in such a way that, according to the amount of cost; will provide care to patients that is beneficial for a large number of patients and focus on achieving the greatest benefit for a large number of them [35]. According to the studies, most ethical theories have accepted various aspects of beneficence and consider it as a basis for creating the most benefit for all patients [19].

The attention and focus of the principle of beneficence in nursing management are also on providing safe, effective, timely, efficient, fair, and patient-centered care combined with accuracy, kindness, and staff collaboration [37]. In such ethical climates, nursing staff is supported by managers in decisions- making For the benefit of patients [67]. With this point of view, the ethical principle of beneficence will undoubtedly be an integral part of the mission of the nursing profession to reduce the pain and suffering of patients [68]. Achieving this requires nurses to be equipped with ethical knowledge and insight [16], and the responsibility of nurses as an ethical commitment for increasing patient’s trust and their families, so that the beneficence applicability will reduce their pain and suffering, especially in end-of-life and palliative care [41].

All patients should be treated with dignity and protected from any possible harm, it is the professional’s moral obligation, to not cause harm [69, 70]. Thus, beneficence and non-maleficence are connected to the patient’s rights, and the safety of care that guarantees care free of danger or risk of injury [71]. Each care program and treatment should provide absolute benefit to the patient, and the nurse’s essential responsibility is to ensure the individual’s safety. As a result, the nurse considers all of the care practices that will be beneficial to the patient before implementing them [72, 73], and in accordance with these principles, they revealed that they valued patient safety, sought to avoid risk and harm, and sought to help cope, regardless of illness or functional impairment [74]. When medical errors are not reported, the principle of beneficence is threatened, as non-reporting prevents other healthcare professionals from accessing relevant information and avoiding similar errors. Ethics and patient safety are intertwined, so, a lack of honesty in communication and a lack of commitment to finding solutions to adverse events is both disrespectful to beneficence and non-maleficence principles, and it compromises patient safety [75].

Truth-telling to the patient as an issue of communication and trust is considered as the applicability of beneficence, which nurses should combine with the patient’s autonomy and balance in their care. As a result, the goal of Truth-telling in health care will be achieving treatment that is effective and based on the patient’s interest [26, 44, 46], and support [16]. In this way, healthcare workers should pay attention to the cultural, social, and ethical differences of patients [16, 26].

In general, it should be noted that the main guideline in ethical judgment about different choices, especially in cases of conflict and dilemmas, is to refer to beneficence, which includes a kind of rational profit in terms of cost-effectiveness regarding the results of the intervention and outcomes for the patient [53].

Studies show that although nurses have good knowledge about nursing ethics, most are not familiar with the principle of beneficence in nursing care and what is good for the patient and for her/his benefit [34, 42]. However, health promotion programs, policies, research, and access to health care have the principle of beneficence at their core [20]. Therefore, considering the importance of the principle of beneficence in nursing care; it is necessary to carry out appropriate studies with the approach of the beneficence concept and different aspects of this important ethical principle.

## Conclusion

The ethical principle of beneficence is considered an important issue and one of the primary values in nursing care, and this is while the results of the present study clarify different aspects of this principle. Lack of acquainted with the definition of the beneficence concept in nursing care is an important challenge that makes it necessary to conduct more studies focusing on the analysis of the concept of the beneficence and developing appropriate instruments in different nursing wards and fields for its measure and application. It seems that conducting clarification of the approach of the principle of beneficence in nursing care can provide positive outcomes for patients to benefit from this principle.

On the other hand, paying attention to the applicability obtained from studies that focus on reducing and predicting pain, fair distribution of resources and care, performing evidence-based care, not harming the patient, telling the truth in the correct situation, performing palliative and ending life care and finally the patient’s safety in nursing care leads to increasing the well-being and health of patients, reducing their mortality and preserving human respect and dignity. Promoting a dignified life by observing the principle of beneficence for patients in nursing care, and paying attention to the principles of autonomy and justice, will ultimately increase patients’ satisfaction. Therefore, it is suggested that more studies should be done focusing on identifying the exact dimensions of the concept of beneficence, developing an appropriate instrument to measure this concept in nursing care, and providing adequate training in this direction.

### Limitations

The limitations of this study include: not searching for articles in languages other than English and Persian, so our search strategies may have under-represented studies in other languages, such as Spanish and Portuguese.

## Data Availability

The datasets used and/or analyzed during the current study are available from the corresponding author upon reasonable request. All requests relating to data should be addressed to rozitacheraghi@gmail.com.
